# Stabilization of ADAM9 by N-α-acetyltransferase 10 protein contributes to promoting progression of androgen-independent prostate cancer

**DOI:** 10.1038/s41419-020-02786-2

**Published:** 2020-07-27

**Authors:** Yung-Wei Lin, Yu-Ching Wen, Chih-Ying Chu, Min-Che Tung, Yi-Chieh Yang, Kuo-Tai Hua, Ke-Fan Pan, Michael Hsiao, Wei-Jiunn Lee, Ming-Hsien Chien

**Affiliations:** 1 Department of Urology, Wan Fang Hospital, Taipei Medical University, Taipei, Taiwan; 2 International Master/PhD Program in Medicine, College of Medicine, Taipei Medical University, Taipei, Taiwan; 3 TMU Research Center of Urology and Kidney (TMU-RCUK), Taipei Medical University, Taipei, Taiwan; 4 Department of Urology, School of Medicine, College of Medicine, Taipei Medical University, Taipei, Taiwan; 5 Graduate Institute of Clinical Medicine, College of Medicine, Taipei Medical University, Taipei, Taiwan; 6 Department of Surgery, Tungs’ Taichung Metro Harbor Hospital, Taichung, Taiwan; 7 Department of Medical Research, Tungs’ Taichung MetroHarbor Hospital, Taichung, Taiwan; 8 Graduate Institute of Toxicology, College of Medicine, National Taiwan University, Taipei, Taiwan; 9 Genomics Research Center, Academia Sinica, Taipei, Taiwan; 10 Department of Medical Education and Research, Wan Fang Hospital, Taipei Medical University, Taipei, Taiwan; 11 TMU Research Center of Cancer Translational Medicine, Taipei Medical University, Taipei, Taiwan; 12 Pulmonary Research Center, Wan Fang Hospital, Taipei Medical University, Taipei, Taiwan; 13 Traditional Herbal Medicine Research Center, Taipei Medical University Hospital, Taipei, Taiwan

**Keywords:** Cancer, Prostate cancer, Cell migration

## Abstract

N-α-Acetyltransferase 10 protein (Naa10p) was reported to be an oncoprotein in androgen-dependent prostate cancer (PCa; ADPC) through binding and increasing transcriptional activity of the androgen receptor (AR). PCa usually progresses from an androgen-dependent to an androgen-independent stage, leading to an increase in the metastatic potential and an incurable malignancy. At present, the role of Naa10p in androgen-independent prostate cancer (AIPC) remains unclear. In this study, in silico and immunohistochemistry analyses showed that Naa10 transcripts or the Naa10p protein were more highly expressed in primary and metastatic PCa cancer tissues compared to adjacent normal tissues and non-metastatic cancer tissues, respectively. Knockdown and overexpression of Naa10p in AIPC cells (DU145 and PC-3M), respectively, led to decreased and increased cell clonogenic and invasive abilities in vitro as well as tumor growth and metastasis in AIPC xenografts. From the protease array screening, we identified a disintegrin and metalloprotease 9 (ADAM9) as a potential target of Naa10p, which was responsible for the Naa10p-induced invasion of AIPC cells. Naa10p can form a complex with ADAM9 to maintain ADAM9 protein stability and promote AIPC’s invasive ability which were independent of its acetyltransferase activity. In contrast to the Naa10p-ADAM9 axis, ADAM9 exerted positive feedback regulation on Naa10p to modulate progression of AIPC in vitro and in vivo. Taken together, for the first time, our results reveal a novel cross-talk between Naa10p and ADAM9 in regulating the progression of AIPC. Disruption of Naa10p–ADAM9 interactions may be a potential intervention for AIPC therapy.

## Introduction

Prostate cancer (PCa) is the second most common cause of cancer-related mortality in the US and Europe. Recently, the incidence of PCa has also been increasing in Asian countries^1^. The progression and growth of PCa were shown to be dependent on androgens; thus, the androgen receptor (AR) plays a crucial role in PCa development. Inhibiting AR signaling with androgen deprivation therapy (ADT) represents the predominant targeted therapy for advanced and metastatic PCa which is sensitive to androgen. Tumor outgrowth during ADT usually represents a transition from androgen-sensitive disease to a more-aggressive and incurable form making ADT ineffective, so called androgen-independent PCa (AIPC) or castration-resistant PCa (CRPC)^2^. Although many approved systemic therapies such as cytotoxic drugs are able to prolong survival of patients with metastatic AIPC, most patients eventually develop primary or acquired resistance^2^. At present, mutations, amplification, overexpression, or post-translational modifications (PTMs) of the AR were reported to activate the AR^3,4^. The AR undergoes a number of PTMs such as phosphorylation, acetylation, methylation, SUMOylation, and ubiquitination that alter its functional activity, including its transcriptional activity, stability, and cellular localization^5^. Most PTMs of the AR identified to date were determined using the full-length AR in androgen-dependent prostate cancer (ADPC) cells^5^. Until to now, the role of protein PTMs and cotranslational modifications (CTMs) in AIPC cells have rarely been investigated.

N-α-Acetyltransferase 10 protein (Naa10p; also known as arrest-defective 1 [ARD1]) is a catalytic subunit of the major N-terminal acetyltransferase (NAT) complex, NatA. This complex can co-translationally mediate the N-α-acetylation of nascent polypeptides such as Ser, Gly, Ala, Val, Thr, and Cys, emerging from ribosomes or post-translationally catalyze acetylation of ε-lysine on internal residues of proteins^6,7^. N-Terminal acetylation occurs in 80–90% of cytosolic human proteins^8^, and this modification appears to be irreversible because no N-terminal deacetylase has been identified to date^9^. By N-α or N-ε acetylating diverse substrate proteins, Naa10p plays critical roles in cell cycle progression and proliferation^10^, apoptosis^11^, autophagy^12^, and cell motility^13,14^ in different cancer types through regulating protein transcriptional activity, stability, and protein–protein interactions. In addition to acetyltransferase (AT)-dependent activity, Naa10p also harbors AT-independent activity to regulate cell behaviors by enhancing activities of its binding partners^15^ or blocking their ability to interact with other proteins^16,17^. To the present, the role of Naa10p in tumor progression is still controversial due to the diverse substrates of Naa10p in different cancers.

In PCa, Naa10p is reported to be more highly expressed in both tumor tissues and related ADPC and AIPC cell lines compared to normal prostatic tissues and prostatic epithelial cell lines. Naa10p is required for AR-mediated transactivation of target genes to further promote proliferation and tumorigenesis of ADPC in vitro and in vivo, respectively^18^. Mechanistically, Naa10p associates with the AR and heat shock protein 90 (HSP90) to induce acetylation at lysine 618 (K618) of the AR, dissociation of AR-HSP90, nuclear translocation of the AR, and ultimately expression of AR target genes and tumorigenesis of ADPC^19^. Although Naa10p was recently shown to participate in controlling proliferation of ADPC through acetylation of the AR, whether Naa10p has an impact on AIPC progression, and the underlying mechanisms of Naa10p in modulating the progression of AIPC are still undefined. In the present study, we showed that Naa10p is functionally involved in the invasion and metastasis of AIPC. Naa10p is more highly expressed in patients with metastatic PCa compared to those with primary PCa. Mechanistic studies revealed that Naa10p is associated with and stabilizes a disintegrin and metalloprotease 9 (ADAM9) to induce the invasive phenotype of AIPC cells, and these phenomena are independent of its acetyltransferase activity. In contrast, ADAM9 also exerts positive feedback regulation on Naa10p expression in AIPC cells. Our findings highlight the potential of targeting the cross-talk between Naa10p and ADAM9 in therapeutic applications against AIPC.

## Materials and methods

### Reagents, antibodies, and DNA plasmids

5α-Dihydrotestosterone (DHT), bicalutamide (CDX), cycloheximide (CHX), MG132, and dimethyl sulfoxide (DMSO) were purchased from Sigma-Aldrich (St. Louis, MO). Fetal bovine serum (FBS) and charcoal-stripped (c)FBS were obtained from Gibco-BRL (Gaithersburg, MD). Other cell culture materials including antibiotics, trypsin-EDTA, medium, and all medium additives were obtained from Corning (Corning, NY). Naa10-V5 was kindly provided by Dr. Johan R. Lillehaug and Dr. Thomas Arnesen. The point mutation located on the AT domain of Naa10p (R82A) was generated by site-directed mutagenesis using an overlapping polymerase chain reaction (PCR) followed by subcloning. Antibodies used for Western blotting were Naa10p (GB-10511, Genesis Biotech, New Taipei City, Taiwan), ADAM9 (sc-23290, Santa Cruz Biotechnology, Santa Cruz, CA), MMP-12 (AB6010, Millipore, Temecula, CA), AR (sc-816, Santa Cruz), prostate-specific antigen (PSA; #2475, Cell Signaling Technology, Danvers, MA), and ubiquitin (ab7780, Abcam, Cambridge, UK). Anti-flag and anti-β-actin antibodies were obtained from Santa Cruz. Polyvinylidene difluoride (PVDF) membranes for Western blotting were purchased from Bio-Rad (Hercules, CA). Unless otherwise specified, other chemicals used in this study were purchased from Sigma Chemical (St. Louis, MO).

### Cell lines and cell culture

The human AIPC cell lines, PC3 and DU145, and ADPC cell line, LNCaP, were obtained from American Type Culture Collection (Manassas, VA). PC-3M cells are a metastasis-derived variant of PC3 cells which exhibit high metastatic ability in prostate xenograft models^20^. PC3 and PC-3M cells were cultured in minimum essential medium (MEM); DU145 cells were cultured in Dulbecco’s modified Eagle medium (DMEM); and LNCaP cells were cultured in RPMI-1640 supplemented with 10% FBS or cFBS and 1% penicillin-streptomycin. The cell lines were grown as adherent monolayer cultures at 37 °C with 5% CO_2_ in a humidified incubator.

### Lentiviral production and infection

The production of lentiviruses was previously described^21^. Briefly, 293T packaging cells (10^6^) were split into 10-cm^2^ dishes. One day later, cells were transfected with 10 μg pWPI-Naa10p, pWPI-Naa10pR82A, or pWPI-control together with 10 μg of pCMVΔR8.91 (the packaging vector) and 1 μg of pMD.G (the envelope vector). After incubation for 5 h, the transfection medium was replaced with fresh culture medium. Two days later, lentivirus-containing medium was collected and further filtered using a 0.45-μm filter, and target cells were infected with fresh lentivirus-containing medium (supplemented with 8 μg/ml polybrene) for 24 h and subjected to different functional assays. Similarly, short hairpin (sh)RNAs for Naa10p (shNaa10p) and ADAM9 (shADAM9) were obtained from the National RNAi Core Facility at Academic Sinica (Taipei, Taiwan). shRNA lentivirus production and target cell infection were performed as described above.

### Cell-viability assay (MTS assay)

PC-3M and DU145 AIPC cells were stably transfected with Naa10p-V5 or its control vector and seeded in 96-well plates (5 × 10^3^ cells/well) containing culture medium with 10% cFBS or FBS, treated with either DHT, CDX, or vehicle for 24, and then subjected to a cell-viability assay (MTS assay; Promega, Madison WI) according to the manufacturer’s instructions. Data were collected from three replicates.

### Plate clonogenic assay

PC-3M and DU145 AIPC cells expressing Naa10p-V5, shNaa10p, or a control vector were seeded in six-well plates at a seeding density of 10^3^ cells/well and incubated for 24 h. Thereafter, the medium was changed every 2 days, and after 10 days of incubation, cells were stained with crystal violet, and colonies were manually counted using ImageJ free software (National Institutes of Health, Bethesda, MD).

### Transwell Matrigel-invasion assay

An in vitro Matrigel-invasion assay was as previously described^22^. Briefly, 3 × 10^4^ PC-3M and DU145 AIPC cells expressing Naa10p-V5, shNaa10p, or a control vector were plated in a Matrigel-coated (BD Biosciences, Bedford, MA) top chamber which contained serum-free medium. Medium supplemented with serum was used as a chemoattractant in the lower chamber. After 24∼48 h of incubation, invading cells on the lower surface of the membrane were fixed with methanol and stained with crystal violet. The number of cells invading through the membrane was counted under a light microscope (×100, three random fields per well).

### Protein lysate preparation and Western blot analysis

Protein lysates were prepared as described previously^23^. Total cellular proteins were determined using a Bio-Rad protein assay kit (Bio-Rad, Hercules, CA). Equal amounts of protein were subjected to sodium dodecylsulfate polyacrylamide gel electrophoresis (SDS-PAGE) or SDS-PAGE gradient gels (BIOTOOLS, New Taipei City, Taiwan) and then electrophoretically transferred to PVDF membranes (Bio-Rad). Membranes were then incubated with indicated primary antibodies and horseradish peroxidase-conjugated secondary antibodies. After washing, blots were incubated with the ECL Western blotting reagent, and chemiluminescence was detected by the chemiluminescence imaging system, MultiGel-21 (TOP BIO, New Taipei City, Taiwan). Image-Pro Plus software (Media Cybernetics, Silver Spring, MD) was used to quantify the density of specific bands.

### Castration of mice

To study the effect of androgen ablation on Naa10p-mediated PCa growth, castration surgery was performed. Age-matched male severe combined immunodeficient (SCID) mice, at 6–8 weeks of age, were anesthetized, and the area around the scrotum was cleaned with Betadine and 70% ethanol. Mice were castrated via a scrotal incision. Briefly, an incision was made at the tip of the scrotum and then the testis, epididymis, vas deferens, spermatic blood vessels, and attached testicular fat pad were pulled out of the incision. A single ligature was placed around the spermatic blood vessels and vas deferens, then severed just below the site of the ligature. The tunica on the contralateral side was similarly penetrated, and the procedure repeated. The scrotum incision was sutured, and castrated mice were put on a heating pad until recovery. The mice were subcutaneously given buprenorphine (0.05 mg/kg) as an analgesic once they began to slightly move around. The animals were allowed to recover from surgery for a week before being used in tumor inoculation experiments.

### In vivo AIPC orthotopic model

Normal or castrated SCID mice were used in assays for tumor growth and metastasis in an orthotopic graft model. All surgical mice received the same anesthetic regimen with a lower abdominal incision. Luciferase-tagged PC-3M or DU145 cells (5 × 10^5^) expressing Naa10p-V5, shNaa10p, shADAM9, shADAM9/Naa10p-V5, or a control vector were suspended in Matrigel and further injected into the prostate using a 30-gauge needle. Luciferase-based, noninvasive bioluminescent imaging and analysis were performed with the Xenogen IVIS-200 system (Xenogen, Alameda, CA). After 42 days, PC-3M-injected mice were sacrificed, and luciferase activities in the excised organs (liver, lung, bone, and pancreas) were further determined using the IVIS-200 system. Metastatic lymph nodes (iliac and sacral) were enumerated at the end of the study. All animal studies were performed according to protocols approved by the Institutional Animal Care and Use Committee of Taipei Medical University (approval number: LAC-2019-0289).

### Transient transfection of DNA

The pReceiver-M14-ADAM9 plasmid was obtained from GeneCopoeia (Rockville, MD). To overexpress ADAM9, AIPC cells were transfected with 3 μg of an empty or ADAM9-expressing vector using Lipofectamine 3000 transfection reagent (Invitrogen, Carlsbad, CA) for 6 h according to the manufacturer’s instructions. At 24 h after transfection, cells were analyzed for invasion and expression of ADAM9.

### RNA preparation and reverse-transcriptase polymerase chain reaction (RT-PCR)

Messenger (m)RNA was isolated using TRizol (Invitrogen, Carlsbad, CA) and amplified by PCR using 2X Taq PCR MasterMix (BIOTOOLS, New Taipei City, Taiwan) as described previously^21^. Primer sequences of ADAD9 were forward 5′-ATTTACTAAGGTGTGCTGGGTC-3′ and reverse 5′- AGGAAGCTACTAGGAGACACAA-3′.

### Co-Immunoprecipitation (Co-IP)

293T cells were co-transfected by ADAM9-flag and Naa10p-V5 for 48 h. These cells were harvested with NETN lysis buffer (NaCl 150 mM, Tris-base 20 mM, NP-40 0.5%, EDTA 1 mM). In total, 2 mg of cell lysates was incubated with Anti-Flag M2® affinity gel (Sigma; A2220) at 4 °C overnight, and washed five times with NETN buffer. To detect the endogenous interaction of ADAM9 and Naa10p, DU145 cells were lysed in NETN buffer, and 2 mg of cell lysates was incubated with an anti-Naa10p antibody overnight at 4 °C followed by a 1-h incubation with 25 μl of immobilized protein A Sepharose beads. The protein complex was also washed with NETN buffer five times. Proteins collected from 293T and DU145 cells were boiled in 5× sample dye and further analyzed by Western blotting.

### Immunohistochemical (IHC) staining

ADAM9 and Naa10p were respectively detected in the established human PCa tissue microarrays (TMAs) PR1921a and PR1921b (US Biomax, Rockville, MD) by IHC staining. Briefly, sections were deparaffinized using a xylene solution and rehydrated using gradient ethanol concentrations. Deparaffinized sections were boiled in a microwave in 0.1 M citric acid buffer (pH 6.0) for antigen retrieval, and endogenous peroxidase activity was inhibited with 0.5% hydrogen peroxide in methanol for 30 min and blocked using 5% normal goat serum in phosphate-buffered saline (PBS). The TMA was then incubated overnight at 4 °C with corresponding primary antibodies for ADAM9 (1:200; orb353511, Biorbyt, Cambridge, UK) and Naa10p antibody (1:200; GB-10511, Genesis Biotech, New Taipei City, Taiwan), followed by washing in PBS and incubation with the secondary antibody at 1:200, for 1 h at room temperature. The TMA was then observed after incubation with a diaminobenzidine (DAB) kit (Boster, Wuhan, China). The stained TMAs were viewed under an Olympus BX50 light microscope (Olympus, Tokyo, Japan), and images were captured by a SPOT digital microscope camera head. To determine the intensity of Naa10p expression, the staining results were scored under a light microscope as described previously^24^.

### Statistical analysis

Values are presented as the mean ± standard deviation (SD). All statistical analyses were performed using the Statistical Package for Social Science software, vers. 16 (SPSS, Chicago, IL). Data were analyzed using Student’s *t*-test when two groups were compared, and *p* values of <0.05 were considered statistically significant.

## Results

### Naa10p expression correlates with metastasis of PCa patients and modulates proliferation, clonogenicity, and invasion of AIPC cells

**Fig. 1:**
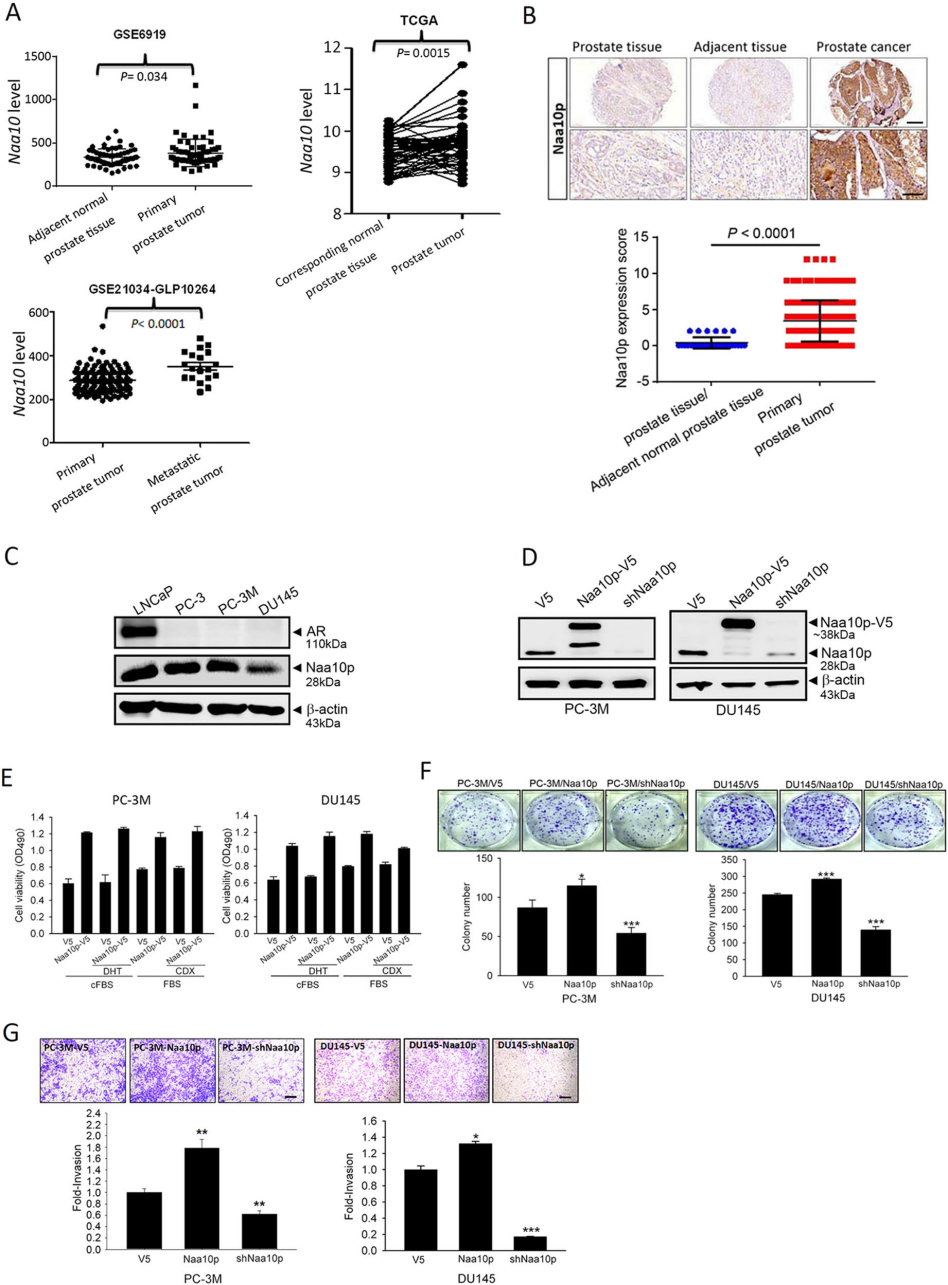
Naa10p expression correlates with metastasis of prostate cancer (PCa) patients and modulates the proliferation, clonogenicity, and invasion of androgen-independent prostate cancer (AIPC) cells. **a** Left panel, *Naa10* gene expression levels in PCa specimens (*n* = 65) and adjacent normal tissue samples (*n* = 63) were measured by Affymetrix oligonucleotide arrays obtained from the GEO (GSE6919). Right panel, Plot depicting an analysis of 52 matched PCa tumor tissues and their corresponding normal tissues (TCGA) revealed higher *Naa10* expression in the tumors. Lower panel, Plot depicting expression levels of *Naa10* in primary (*n* = 131) and metastatic (*n* = 19) PCa specimens. The plot was made using GSE21034. **b** Naa10p protein expression levels in PCa specimens (*n* = 160) and adjacent normal tissue samples or normal prostate tissue samples (*n* = 30) were measured by IHC staining. Scale bars of upper panel and lower panel are 200 μM and 100 μM, respectively. **c** Protein expression levels of Naa10p and the androgen receptor (AR) in ADPC (LNCaP) and AIPC (PC-3, PC-3M, DU145) cell lines. **d** Western blot analysis of Naa10p expressions in PC-3M (left) and DU145 (right) cells expressing Naa10p-V5 or Naa10p shRNA. **e** PC-3M or DU145 cells overexpressing Naa10p-V5 were cultured either in the regular medium with 10 µM bicalutamide (CDX), or medium with charcoal-stripped fetal bovine serum (cFBS) and 10 nM dihydrotestosterone (DHT) for 24 h. Changes in cell viability were determined by MTS assays. **f, g** PC-3M and DU145 cells were infected with a lentivirus carrying Naa10p-V5, Naa10p shRNA, or their respective controls. In vitro tumorigenicity and invasive ability of AIPC cells were determined by counting the colonies formed (**f**) and by a Matrigel-invasion assay (**g**) Scale bar, 100 μM. Representative micrographs of the invasion (100×) and colony-forming assays are shown in the upper panel. Lower panel: Data are presented as the mean ± SD of at three independent experiments. *p < 0.05; **p < 0.01; ***p < 0.001, compared to the control group.

Naa10p was recently reported to promote cell growth through upregulating AR activity in PCa cell lines with an intact AR^18^. The clinical relevance of Naa10p in PCa was analyzed using the GEO and TCGA databases. Significantly higher levels of *Naa10* transcripts were observed in tumors compared to normal tissues (GSE6919) (Fig. 1a, left panel, *p* = 0.034). Moreover, in N/T paired PCa cohort from TCGA, significantly higher *Naa10* transcripts were also observed in tumors compared to corresponding normal tissues (Fig. 1a, right panel, *p* = 0.0015). Furthermore, expressions of *Naa10* transcripts were significantly higher in metastatic tumors than in primary PCa tumors (GSE21034) (Fig. 1a, lower panel, *p* < 0.0001). In addition to in silico analysis, we analyzed Naa10p protein expression in a PCa TMA and observed that Naa10p expression was enriched in PCa tissues, but was very low in normal prostate tissues (Fig. 1b). These data imply that Naa10p may play a critical role in the progression of PCa. Similar to a previous study^18^, we actually observed that overexpression of Naa10p can promote AR activity as indicated by PSA upregulation in ADPC LNCaP cells (Fig. S1). In contrast to LNCaP cells harboring an intact AR, PC3, PC-3M, and DU145 PCa cells with an undetectable AR also expressed Naa10p (Fig. 1c). Herein, we further investigated the effect of Naa10p expression on cell behaviors of PC-3M and DU145 cells through overexpression and knockdown of Naa10p in these cells (Fig. 1d). We first determined the impact of changing Naa10p expression on proliferative rates of PC-3M and DU145 cells in the presence and absence of androgen. Overexpressing V5-tagged Naa10p in PC-3M cells promoted cell proliferation both in regular medium with the anti-androgen, bicalutamide (CDX), and medium with cFBS and the active androgen, DHT (Fig. 1e, left panel). Among PC-3M cells treated with or without CDX and DHT, respectively, in regular medium and cFBS medium, proliferation-promoted effects of Naa10p were all similar (Fig. 1e, left panel). Collectively, these results suggest that Naa10p expression may regulate PCa cell proliferation independent of the androgen-AR axis. Enhanced androgen-independent cell proliferation by overexpressing Naa10p was also observed in another AIPC cell line, DU145 (Fig. 1e, right panel). In addition to the proliferative rate, the colony-forming and invasive abilities of control, Naa10p-overexpressing, and Naa10p-knockdown cells were next estimated with plate clonogenic and transwell invasion assays, respectively. As shown in Fig. 1f, g, overexpression and knockdown of Naa10p respectively increased and decreased the colony-forming and invasive abilities of PC-3M and DU145 cells.

### Naa10p promotes tumorigenicity and the metastatic ability of AIPC in an animal model with or without castration

**Fig. 2:**
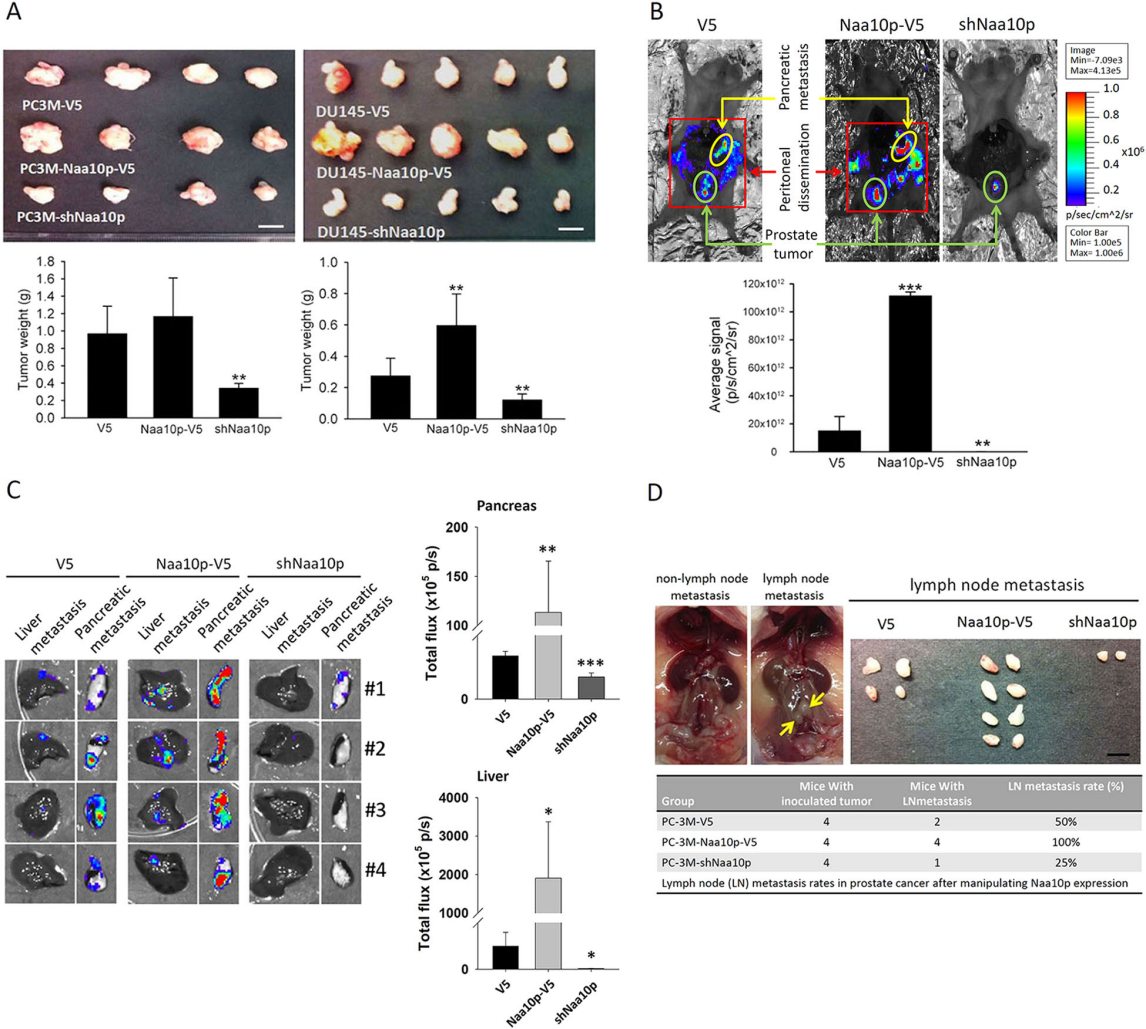
Naa10p promotes androgen-independent prostate cancer (AIPC) growth and metastasis in an orthotopic mouse model. Male NOD/SCID mice were orthotopically injected with luciferase-tagged and Naa10p-overexpressing or Naa10p-knockdown PC-3M and DU145 cells. The tumor progression was monitored by bioluminescence imaging. At 42 days after tumor implantation, animals were sacrificed, and tumor specimens were collected. **a** Upper panel, Gross appearance of orthotopic tumors. Lower panel, Average tumor weight of each group is shown. **p < 0.01, compared to the control group. **b** Upper panel, Cancer metastasis of PC-3M xenografts including proximal invasion and distal metastasis was imaged with bioluminescence at the end of the study. Lower panel, Quantitative analysis of imaging signal intensity (photons/s/cm^2^/sr) with the mean signal for each group indicated. **p < 0.01; ***p < 0.001, compared to the control group. **c** Livers and pancreases were isolated and examined at the end of this spontaneous metastasis assay. Left panel, Cancer metastasis from the prostate to the liver and pancreas was imaged with bioluminescence. Right panel, Pancreas and liver metastatic signal intensities were bioluminescently captured at the end of the study, with the mean signal for each group (*n* = 4) indicated. *p < 0.05; **p < 0.01; ***p < 0.001, compared to the control group. **d** Occurrence of regional lymph node (sacral and iliac) metastasis in NOD/SCID mice injected with PC-3M/shNaa10p, PC-3M/Naa10p-V5, or control PC-3M cells. Yellow arrows in the left panel indicate iliac lymph node metastasis. Right and lower panels show the appearance, number, volume, and frequency of regional lymph nodes.

To further investigate the in vivo effects of Naa10p on AIPC tumor progression, we established an orthotopic PCa-bearing model by transplanting luciferase-tagged cells, PC-3M/DU145-mock-luciferase, PC-3M/DU145-Naa10p-luciferase, or PC-3M/DU145-shNaa10p-luciferase, into NOD-SCID mice that had or had not undergone surgical castration. In non-castrated mice, control DU145 cells orthotopically injected into NOD-SCID mice formed smaller and larger tumors than those in mice respectively injected with DU145/Naa10p-V5 cells and DU145/shNaa10p cells according to evidence from tumor weight measurements at the end of the experiment (6 weeks after cell injection) (Fig. 2a, right panel). Similar tumor-modulating effects of Naa10p were also observed in a PC-3M xenograft model (Fig. 2a, left panel). To further validate if the tumor-modulating effects of Naa10p actually were through an androgen-independent pathway, castrated mice were used. In castrated mice, Naa10p-knockdown in PC-3M cells also attenuated tumor growth compared to the control group (Fig. S2, right panel). In contrast, the tumorigenic ability was enhanced in Naa10p-overexpressing DU145 cells (Fig. S2, left panel). In addition to tumorigenesis, the effect of Naa10p on tumor metastasis was monitored by bioluminescence imaging. Formation of spontaneous metastasis in the orthotopic PC-3M-implanted model was, respectively, enhanced and suppressed by overexpression and knockdown of Naa10p in PC-3M cells according to evidence from ex vivo photon imaging (Fig. 2b). After the organs were further excised, ex vivo imaging of the pancreases and livers respectively showed significantly higher and lower photon intensities in PC-3M/Naa10p-V5- and PC-3M/shNaa10p-injected mice compared to control mice (Fig. 2c). Lymph node (LN) metastasis is a well-recognized route of PCa dissemination^25^. We further determined the frequency of regional LN metastasis (iliac and sacral) among PC-3M/V5-, PC-3M/Naa10p-V5-, and PC-3M/shNaa10p-injected mice. Higher (100%) and lower (25%) incidences of LN metastasis were respectively observed in PC-3M/Naa10p-V5- and PC-3M/shNaa10p-injected mice compared to PC-3M/V5-injected mice (50%) (Fig. 2d).

### Naa10p acts as a post-translational regulator to stabilize the protease ADAM9 in AIPC cells

**Fig. 3:**
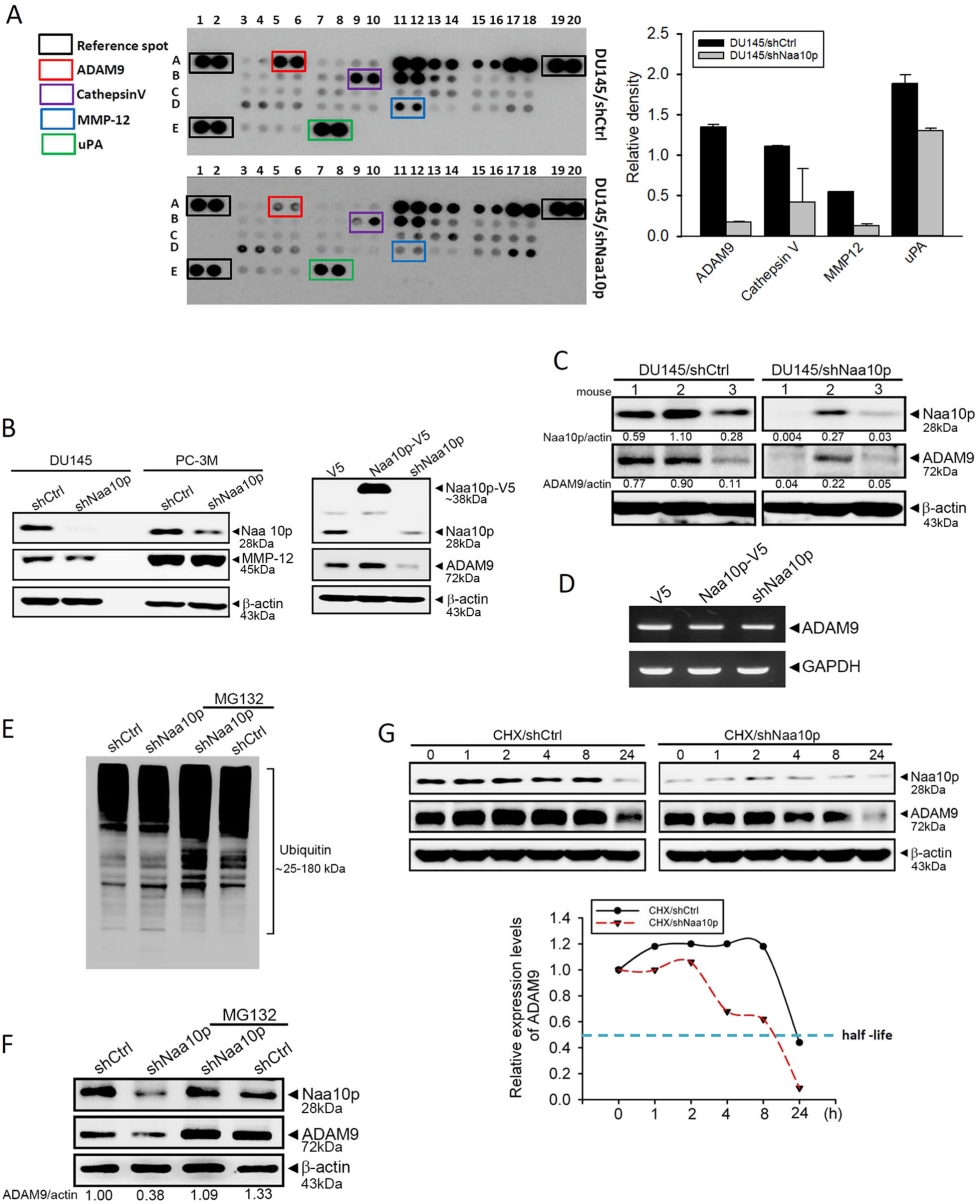
Naa10p acts as a post-translational regulator to stabilize protease ADAM9 in androgen-independent prostate cancer (AIPC) cells. **a** Differential expressions of proteases in cell lysates from DU145/shCtrl and DU145/shNaa10p cells. An antibody array (R&D Systems) detecting 34 different proteases was used to evaluate protease expressions. The left panel shows representative array blots. The right panel shows a quantitative analysis of the protease array using a densitometer. Values are presented as the mean ± SD. *n* = 2. **b, d** PC-3M or DU145 cells expressed shNaa10p, Naa10p-V5, or their respective control as indicated. Cell protein and RNA were extracted, and MMP-12, ADAM9, or Naa10p expression levels were determined by Western blotting (**b**) and an RT-PCR (**d**), respectively. **c** Protein lysates from DU145 orthotopic xenografts with or without Naa10p depletion were subjected to a Western blot analysis to determine ADAM9 expression levels. **e**, **f** Naa10p-depleted and control DU145 cells were treated with or without 10 μM MG-132 for 6 h, and then proteins were extracted. The level of total poly-ubiquitinated proteins (**e**), ADAM9, Naa10p, and β-actin proteins (**f**) were analyzed by Western blotting. Quantitative results of ADAM9 protein levels, which were adjusted to the β-actin protein level. **g** DU145/shCtrl and DU145/shNaa10p cells were treated with 35 μM cycloheximide at different time points, and ADAM9 expression was determined by Western blotting. Quantitative results of ADAM9 protein levels are shown at the bottom.

Proteases are enzymes that cleave or split proteins, and they are known to play important roles during tumor growth, invasion, and metastasis^26^. We next performed proteomic screening with a human protease array (Fig. 3a, left panel) to investigate the effect of Naa10p on protease expressions. Among 34 proteases, four proteases including ADAM9, cathepsin V, MMP-12, and uPA were all downregulated in Naa10p-depleted DU145 cells compared to control cells, and ADAM9 and MMP-12 were the two highly downregulated ones (Fig. 3a, right panel). In addition, a Western blot analysis was performed to further validate whether Naa10p can modulate ADAM9 or MMP-12 expression in AIPC cells. The downregulation of MMP-12 was only observed in Naa10p-depleted Du145, but not in Naa10p-depleted PC-3M cells (Fig. 3b). In contrast, ADAM9 expression was respectively decreased and increased after Naa10p knockdown and overexpression in both cell lines. (Fig. 3b, Fig. S3). Similar to the in vitro results, we observed that ADAM9 expression in DU145 xenografts dramatically decreased in the Naa10p-knockdown group compared to the control group. Expression levels of ADAM9 and Naa10p were positively correlated in these xenografts (Fig. 3c). We next investigated how Naa10p affects ADAM9 protein expression. Data from an RT-PCR showed that the ADAM9 mRNA level was not affected by Naa10p overexpression or knockdown in DU145 and PC-3M cells (Fig. 3d, Fig. S4), indicating that Naa10p might regulate ADAM9 expression through translational or post-translational regulation. After knockdown of Naa10p in DU145 cells, an increase of ubiquitinated proteins was observed (Fig. 3e), implying that Naa10p depletion-induced decrease of ADAM9 protein level might be due to the ubiquitin-proteasome-mediated degradation of ADAM9^27^. To further confirm this issue, the proteasome inhibitor, MG-132, was used, and we found that pretreatment of DU145 cells with MG-132 could prevent ADAM9 degradation after Naa10p-knockdown (Fig. 3f). We next determined whether Naa10p can affect the protein half-life of ADAM9 using the protein synthesis inhibitor, CHX. After CHX treatment, the ADAM9 protein decreased faster in DU145/shNaa10p cells than in DU145/shCtrl cells (Fig. 3g). Taken together, these results indicated that Naa10p may act as a post-translational regulator of ADAM9 through preventing ADAM9 from proteasome-dependent protein degradation.

### ADAM9 plays a critical role in Naa10p-modulated invasive ability in AIPC cells

**Fig. 4:**
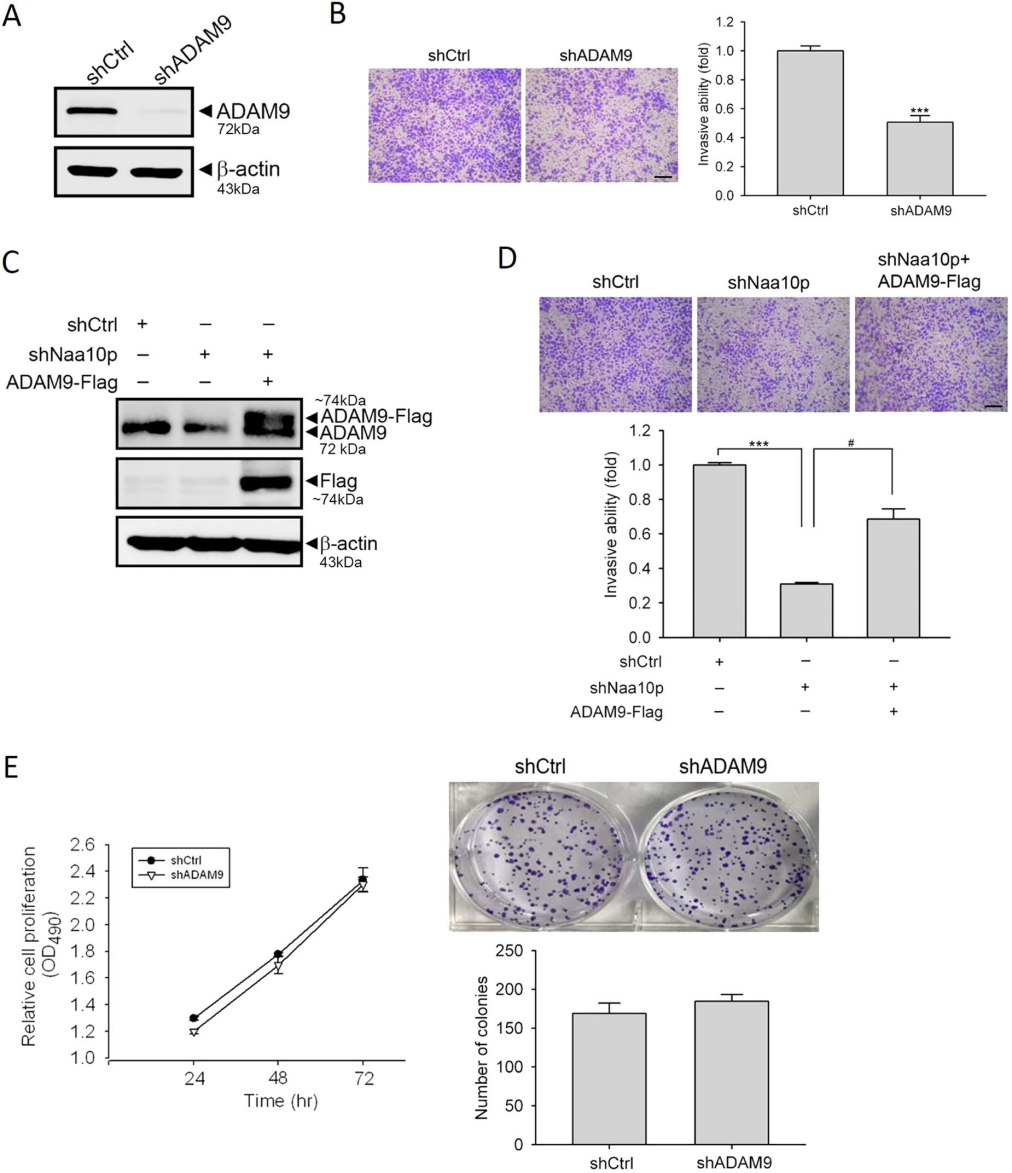
ADAM9 plays a critical role in the Naa10p-modulated invasive ability of androgen-independent prostate cancer (AIPC) cells. **a, b** DU145 cells were infected with a lentivirus carrying ADAM9 small hairpin (sh)RNA or shGFP (shCtrl) and subjected to Western blot (**a**) and Matrigel-invasion assays (**b**) Scale bar, 100 μM. Multiples of differences are presented as the mean ± SD of three independent experiments. ***p < 0.001, compared to the control group. **c**, **d** An ADAM9-expressing plasmid (ADAM9-flag) was transfected into Naa10p-knockdown cells as indicated and subjected to Western blot (**c**) and Matrigel-invasion assays (**d**) Scale bar, 100 μM. The overexpression efficiency of ADAM9 is shown according to a Western blot analysis using a flag antibody. Multiples of differences are presented as the mean ± SD of three independent experiments. ***p < 0.001, compared to the control group; ^#^*p* < 0.05, compared to the Naa10p knockdown only group. **e** The proliferative ability of DU145 cells infected with shGFP or shADAM9 were analyzed by an MTS assay (left panel) and colony-forming assay (right panel). Values are presented as the mean ± SD of three independent experiments.

ADAM9 harbors extracellular matrix (ECM) proteolytic activity and was reported to be overexpressed in PCa tissues, particularly in patients who had received ADT, and is known to be associated with PCa progression, radiosensitivity, chemosensitivity, and biochemical recurrence^28–31^. We next determined the functional role of ADAM9 in AIPC by knocking-down ADAM9 in DU145 cells. The knockdown efficiency of ADAM9-specific shRNA was detected by a Western blot analysis (Fig. 4a). After knocking-down ADAM9, the invasive abilities of DU145 cells were significantly downregulated compared to control cells (Fig. 4b). To further investigate the role of ADAM9 expression in the Naa10p-regulated cell-invasive ability, we re-expressed ADAM9 in Naa10p-knockdown DU145 cells by transfecting an ADAM9-expressing plasmid (Fig. 4c). Overexpression of ADAM9 dramatically reversed the invasion suppression induced by Naa10p-knockdown in DU145 cells, suggesting the dependence on ADAM9 of the Naa10p-regulated cell-invasive ability (Fig. 4d). In contrast to its invasive ability, depletion of ADAM9 did not affect the proliferation or clonogenicity of DU145 or PC-3M cells (Fig. 4e, Fig. S5).

### Naa10p associates with ADAM9, and its AT activity is not necessary to regulate ADAM9 stability and invasive ability in AIPC cells

**Fig. 5:**
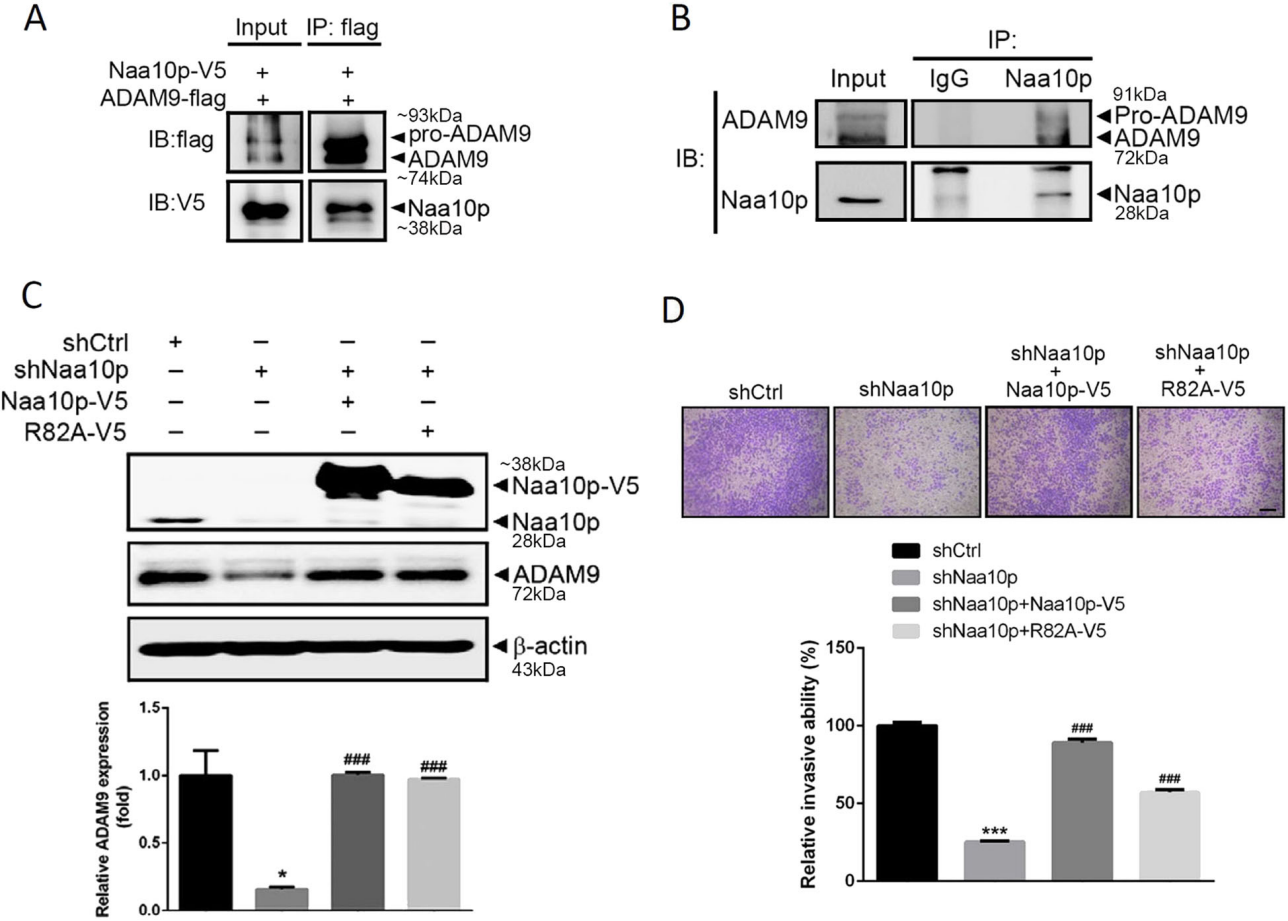
Naa10p associates with ADAM9, and its acetyltransferase activity is not essential for regulating ADAM9 stability or the cell-invasive ability. **a** 293 T cells were transfected with a flag-ADAM9-expressing vector and a V5-Naa10p-expressing vector. Cell lysates were immunoprecipitated (IP) with a flag antibody and then subjected to immunoblotting with the indicated antibodies. **b** The immunocomplex was precipitated from DU145 cell lysates with a Naa10p antibody and analyzed by Western blotting to detect Naa10p and ADAM9 associations. A normal IgG antibody was used as an IP control, and 10% whole-cell lysate was used as the input. **c, d** Naa10p-V5, R82A-V5, shNaa10p, and a control vector were respectively overexpressed in DU145 cells as indicated. Expression levels of ADAM9 and Naa10p were detected by a Western blot analysis. β-Actin was used as a loading control. (**c**). Cells were also subjected to a transwell invasion assay to determine their invasive abilities (**d**) Scale bar, 100 μM. Multiples of differences are presented as the mean ± SD of three independent experiments. ***p < 0.001, compared to the control group; ^###^*p* < 0.001, compared to the Naa10p-knockdown only group.

We next tested whether Naa10p regulates ADAM9 stability through binding with ADAM9. Co-IP experiments of 293T cells co-transfected with ADAM9-flag and Naa10p-V5 revealed that Naa10p interacted with ADAM9 (Fig. 5a). Endogenous interactions between Naa10p and ADAM9 were also observed in DU145 cells (Fig. 5b). Naa10p was reported to regulate its target proteins and cellular functions in both AT activity-dependent and AT-independent manners^6^. To further investigate whether AT activity of Naa10p is needed to modulate ADAM9 stability and invasive ability, an Naa10p-R82A mutant harboring low AT activity^16^ was used. Similar to wild-type Naa10p, overexpression of R82A significantly reversed the inhibitory effect of Naa10p-knockdown on ADAM9 expression (Fig. 5c) and the invasive ability of DU145 cells (Fig. 5d). These results suggest that Naa10p regulates ADAM-9-mediated cell invasion in AIPC cells through a mechanism independent of its intrinsic AT activity.

### Feedback regulation of ADAM9 on Naa10p expression modulates progression of AIPC cells in vitro and in vivo

**Fig. 6:**
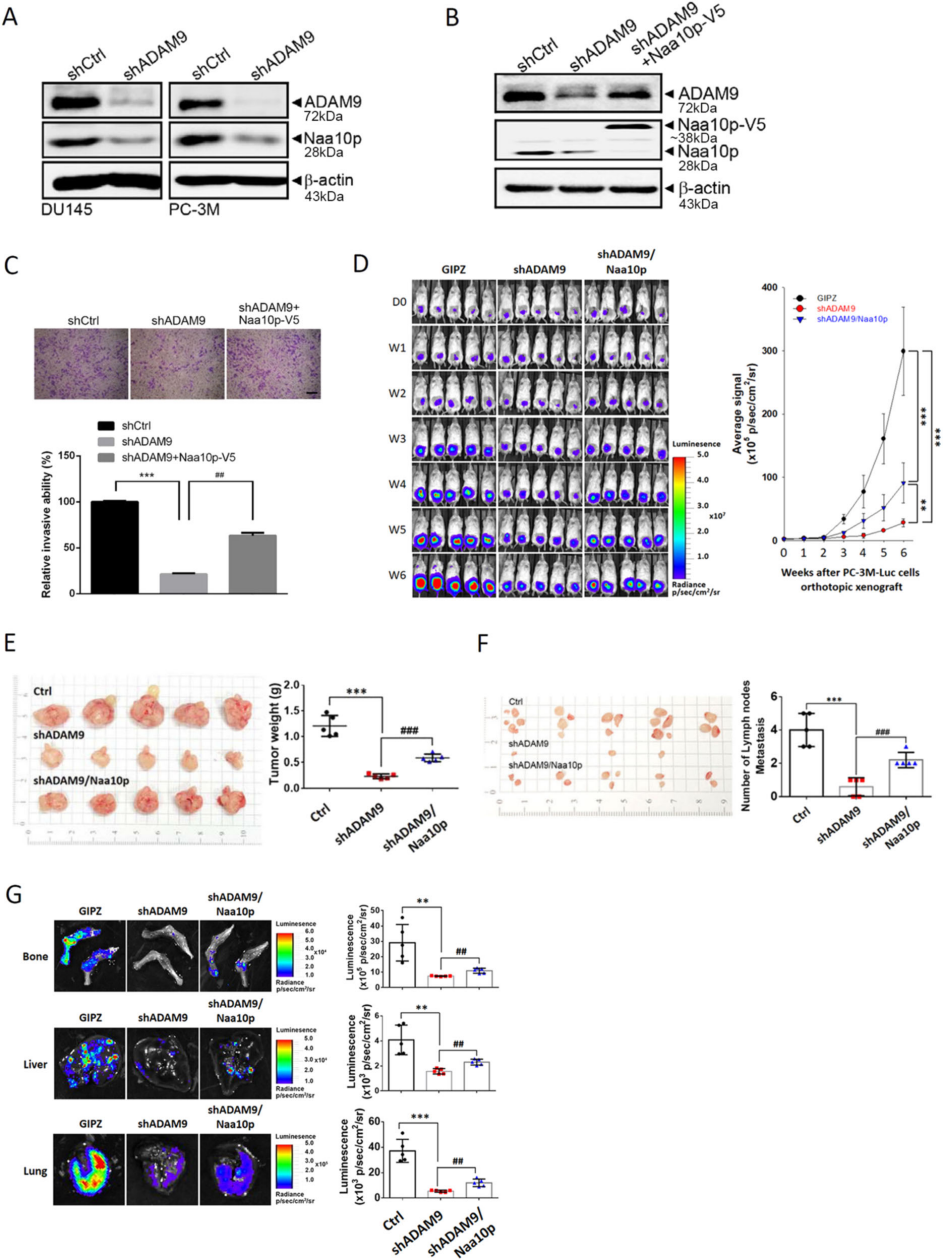
ADAM9 exerts positive feedback regulation on Naa10p to promote progression of androgen-independent prostate cancer (AIPC) cells in vitro and in vivo. **a**–**c** AIPC cells overexpressed shADAM9, shADAM9 + Naa10p-V5, or the control vector as indicated and were subjected a Western blot analysis (**a** and **b**) and Matrigel-invasion assay (**c**) Scale bar, 100 μM. Multiples of differences are presented as the mean ± SD. ***p < 0.001, compared to the control group; ^##^*p* < 0.01, compared to the Naa10p-knockdown only group. **d** Left panel, Male NOD/SCID mice were orthotopically injected with luciferase-tagged and ADAM9-depleted (shADAM9) PC-3M cells or ADAM9-depleted and Naa10p-overexpressing PC-3M cells (*n* = 5). Whole-body bioluminescence imaging was conducted at different time points after injecting cells into mice. Right panel, Quantitative analysis of Xenogen imaging signal intensity (photons/s/cm^2^/sr) every week. **p < 0.01, ***p < 0.001. **e** Left panel, tumors were dissected and photographed after 6 weeks (left panel), and the average tumor weight in each group is given (right panel). **f** Left panel, macroscopic analysis of regional lymph nodes. The appearance, number, and volume of regional lymph nodes were photographed and enumerated after removal. **g** Left panel, Representative ex vivo bioluminescence imaging of metastatic sites including bone, liver, and lung at the end of this spontaneous metastasis assay. Signal intensities of metastatic organs were imaged with bioluminescence at the end of the study, with the mean signal for each group indicated. **e**–**g** Right panel, Data are presented as the mean ± SD. **p < 0.01, ***p < 0.001 compared to the control group. ^##^*p* < 0.01, ^###^*p* < 0.001 compared to the ADAM9-knockdown only group.

In contrast to the Naa10p-ADAM9 axis in regulating AIPC progression, we surprisingly found that knockdown of ADAM9 also reduced Naa10p expression in DU145 and PC-3M cells (Fig. 6a), suggesting that ADAM9 might exert feedback regulation on Naa10p expression in AIPC cells. Moreover, overexpression of Naa10p dramatically reversed suppression of the invasive ability induced by ADAM9-knockdown in PC-3M cells, suggesting the dependence on Naa10p of the ADAM9-regulated cell-invasive ability (Fig. 6b, c). In vivo, we observed that control PC-3M cells (PC-3M/shCtrl) orthotopically injected into SCID mice produced larger tumors than did PC-3M/shADAM9 cells injected into mice after 6 weeks, as revealed by photon emission detection. Re-expression of Naa10p in PC-3M/shADAM9 cells significantly restored tumor growth abilities (Fig. 6d). Changes in mean tumor weights from removed xenografts also revealed similar trends with the tumor size detected by in vivo images (Fig. 6e). PCa metastases usually occur in lymph nodes, bones, liver, and lungs^32^. The mean numbers of metastatic lymph nodes in PC-3M/shADAM9 mice were significantly lower compared to those in control mice (Fig. 6f). From ex vivo imaging of different tissues, we also observed that ADAM9 depletion significantly attenuated the bone, liver, and lung metastatic potential of PCa (Fig. 6g). All of these phenomena observed in ADAM9-depleted cells could be significantly reversed when cells re-expressed Naa10p (Fig. 6f, g). These results suggest that ADAM9 exerts positive feedback regulation on Naa10p expression to modulate AIPC progression.

## Discussion

At present, treatment of patients with metastatic (m)AIPC remains a significant clinical challenge. Over the past several years, taxanes still remain the only form of chemotherapy for mAIPC treatment, but most patients eventually develop taxane resistance. Until recently, patients with mAIPC had limited treatment options after taxane chemotherapy^33^. Hence, metastasis and therapeutic resistance are the major causes of failure of mAIPC treatment, and new therapeutic strategies for mAIPC are urgently needed. ADAM9 is a metzincin cell-surface protease involved in diverse cellular processes such as proliferation, cell migration, ECM binding, ectodomain shedding, and cell–cell interactions^34^. ADAM9 was found to be overexpressed in several solid tumors. Actually, we also observed that ADAM9 is more highly expressed in PCa tissues than in adjacent normal tissues or normal prostate tissues in the PCa TMA cohort (Fig. S6). In PCa, ADAM9 was reported to be significantly associated with shortened PSA relapse-free survival^31^. Moreover, ADAM9 in combination with urinary vascular endothelial growth factor (VEGF) may be correlated with the risk of PCa recurrence and death in patients who underwent ADT^35^. In mAIPC cell lines and an in vivo xenograft model, depletion of ADAM9 enhanced radiosensitivity and chemosensitivity of mAIPC cells, and suppressed tumor bone metastasis in vivo^28,29^. According to these results, ADAM9 might be a druggable target for treatment of mAIPC. Although ADAM9 may be a druggable target for PCa, ADAM9 is also involved in regulating several physiological processes in normal cells, including fertilization, myogenesis, migration, and cell survival^34^. Hence, use of an ADAM9 inhibitor for PCa treatment might exert unpredictable toxicities, just like other MMP inhibitors which were reported to induce intolerable musculoskeletal pain and inflammation in cancer treatment clinical trials^36^. Recently, several studies reported that a number of micro (mi)RNAs can suppress tumor progression through destabilizing ADAM9 mRNA and attenuating translation. For example, miR126 was demonstrated to exhibit anticancer effects in PCa and breast cancer through targeting ADAM9^30,37^. This direct regulation of ADAM9 by miRNAs creates a potential niche for development of miRNA-based therapies in cancer treatment^34^. However, major challenges for miRNA-based therapies are issues of potential off-target effects and safety^38^. Hence, the clinical success for targeting ADAM9, they should need to be highly selective and able to accumulate in cancer tissues without eliciting systemic toxic effects. In this study, we identified that ADAM9 protein stability is modulated by Naa10p, which is highly expressed in PCa, but rarely expressed in normal prostate tissues^18^. Our findings indicate that the potential of targeting of the Naa10p-ADAM9 regulatory axis might be a novel therapeutic strategy against AIPC.

Naa10p was recently demonstrated to be a downstream target gene of the androgen-AR axis and functions as a tumor promoter in ADPC through interacting and acetylating the AR and activating AR-mediated gene expressions such as of PSA^18^. Although the highest levels of Naa10p were found in AR-containing PCa cell lines, levels of Naa10p in AR-null cell lines were also relatively higher than those in normal prostatic epithelial cell lines^18^, suggesting other sets of genes in androgen-AR axis-independent PCa cells are regulated by Naa10p through a non-AR-dependent signaling pathway. Indeed, our present results showed that Naa10p can promote progression of AR-null AIPC cells through inducing ADAM9 stabilization and upregulation. At this time, the mechanism influencing Naa10p expression in AR-null AIPC is unknown. Our results first showed that ADAM9 exerts positive feedback regulation on Naa10p expression to modulate AIPC progression. Bone morphogenetic protein 2 (BMP2), a bone formation stimulator, was reported to induce Naa10p and ADAM9 during osteogenic differentiation^39,40^. BMP2 was also reported to promote invasive and proliferative abilities in ADPC and AIPC cells^41,42^. Cross-talk among Naa10p, ADAM9, and BMP2 in regulating progression of AIPC warrants further investigation. Moreover, Bluemn et al. recently indicated that the independence of the AR pathway in AIPC was associated with elevated autocrine fibroblast growth factor (FGF) signaling, elevated FGF receptor (FGFR), and mitogen-activated protein kinase (MAPK) pathway activity in mAIPC^43^. In a mouse model of PCa, ADAM9 was shown to play a critical role in tumor progression which was attributed to the ability of ADAM9 to cleave and release EGF and FGFR2 from cells^44^. The correlation between Naa10p-ADAM9 and FGF-FGFR in AIPC also need to be further dissected in the future.

The effects of Naa10p on cell invasiveness and metastasis are diverse and complicated in different cancer types. In non-small cell lung cancer (NSCLC), Naa10p was found to be expressed at a lower level in metastatic lymph nodes compared to primary tumors^16^. The expression level of Naa10p in breast cancer was reported to be inversely correlated with lymph node metastasis^17^. In contrast, patients with hepatocellular carcinoma (HCC) harboring higher Naa10p expression showed more-frequent microvascular invasion and poorer survival^45^. With osteosarcomas, higher Naa10p expression was observed in patients with metastasis than in patients without metastasis^13^. Naa10p was demonstrated to regulate cancer cell behavior both dependent on and independent of its catalytic activity^6^. In a catalytic activity-independent manner, Naa10p interacted with p21-activated kinase-interacting exchange factor (PIX) to block its downstream Rac1/Cdc42 pathway and suppressed NSCLC metastasis^16^. In breast cancer, Naa10p inhibited metastasis through binding to the transcription factor, signal transducer and activator of transcription 5a (STAT5a), and decreasing STAT5a-mediated ID1 expression^17^. In a catalytic activity-dependent manner, Naa10p was directly associated with and acetylated MMP-2 to maintain MMP-2 protein stability and promote metastasis of osteosarcomas^13^. In fibrosarcoma cells, Naa10p bound to and acetylated myosin light chain kinase (MLCK) on K608 to suppress MLCK activity and MLCK-mediated invasive ability^14^. These results indicated that targets with which Naa10p interacts are diverse in different cancer types, and distinct activities of Naa10p in modulating its associated proteins also mediate diverse cellular function of cancer cells. Actually, our current data showed that knockdown of Naa10p can inhibit cell invasiveness and downregulate several proteases including ADAM9, cathepsin V, MMP-12, and uPA in DU145 AIPC cells. Overexpression of ADAM9 significantly, but not completely reversed the invasion suppression induced by knockdown of Naa10p in DU145 cells. In contrast, knockdown of ADAM9 also reduced Naa10p expression in AIPC cells and overexpression of Naa10p just partially, but not completely reversed the invasion/metastasis suppression induced by ADAM9-knockdown in DU145 cells in vitro and in vivo. Collectively, these results suggest that ADAM9 is not the only one target involved in Naa10p-modulated AIPC progression, but the roles of other Naa10p’s targets such as other proteases or binding factors in AIPC progression are needed to be further identified.

**Fig. 7:**
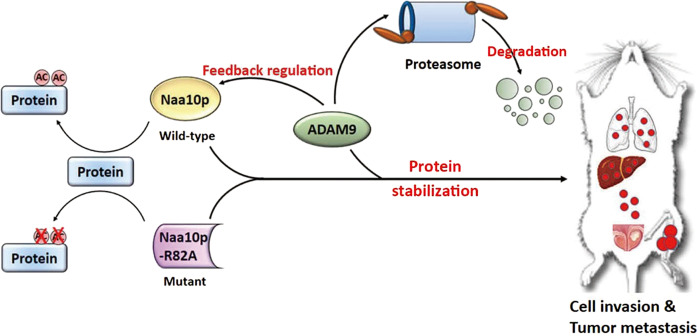
Schematic presentation depicting the cross-talk between Naa10p and ADAM9 in promoting androgen-independent prostate cancer (AIPC) progression. In AIPC cells, Naa10p was highly expressed and associated with ADAM9 to maintain its protein stability through AT-independent manner thus contributing to cell invasion and metastasis. Upregulation of ADAM9 can further exert positive feedback regulation on Naa10p expression.

In this study, we provided a novel mechanism to address how Naa10p regulates tumor progression in AIPC. Our data showed that high expression of Naa10p is associated with increased metastasis in PCa patients. Naa10p may function as a promoter of AIPC metastasis by stabilizing ADAM9 through AT-independent manner. ADAM9 is a well-documented oncoprotein in AIPC and exerts positive feedback regulation on Naa10p (Fig. 7). These results reveal a novel convergence between Naa10p and ADAM9 in regulating the progression of AIPC and provide possible therapeutic relevance for the development of anticancer agents. To the best of our knowledge, we believe that this is the first work showing that Naa10p can regulate PCa progression independent of the androgen-AR axis.

## Supplementary information

Supplementary Figure Legends

Figure S1

Figure S2

Figure S3

Figure S4

Figure S5

Figure S6
