# Supplementary material for: Stabilization of ADAM9 by N-α-acetyltransferase 10 protein contributes to promoting progression of androgen-independent prostate cancer
Source: Cell Death Dis. 2020 Jul 27;11(7):591. doi: 10.1038/s41419-020-02786-2 (PMC7385149; doi:10.1038/s41419-020-02786-2)
Supplement: Supplementary file 1 — Supplementary Figure Legends [file 41419_2020_2786_MOESM1_ESM.docx]

**Supplementary Figure Legends**

**Figure S1.** Effect of Naa10p overexpression on prostate-specific antigen (PSA) expression in LNCaP androgen-dependent prostate cancer (ADPC) cells. Western blot analysis of PSA expressions in LNCaP cells overexpressing Naa10p-V5. Quantitative results of PSA protein levels, which were adjusted to β-actin protein levels.

**Figure S2.** Effect of Naa10p on tumor growth in a DU145 and PC-3M xenograft model that received surgical castration. One week after receiving surgical castration, male NOD/SCID mice were orthotopically injected with Naa10p-overexpressing DU145 (*n* = 5 in each group) and Naa10p-knockdown PC-3M (*n* = 3 in each group) cells. Upper panel, Gross appearance of orthotopic tumors 42 days after tumor cell injection. Lower panel, Average tumor weight of each group is shown. ** *p* < 0.01, compared to the control group.

**Figure S3.** Naa10p modulates ADAM9 protein expression in PC-3M androgen-independent prostate cancer (AIPC) cells. PC-3M cells expressed shNaa10p, Naa10p-V5, or their respective control as indicated. ADAM9 and Naa10p protein expression levels were determined by a Western blot analysis. Quantitative ADAM9 levels were adjusted to β-actin levels.

**Figure S4.** Effect of Naa10p on ADAM9 mRNA expression in PC-3M androgen-independent prostate cancer (AIPC) cells. PC-3M cells expressed shNaa10p, Naa10p-V5, or control vector as indicated. ADAM9 mRNA expression levels were determined by an RT-PCR analysis. Quantitative ADAM9 levels were adjusted to GAPDH levels.

**Figure S5.** Effects of ADAM9 on the proliferative and colony-forming abilities of PC-3M androgen-independent prostate cancer (AIPC) cells. The proliferative and colony-forming abilities of PC-3M cells infected with small hairpin (sh)GFP (shCtrl) or shADAM9 were respectively analyzed by MTS (left panel) and colony-forming (right panel) assays. Values are presented as the mean ± SD of three independent experiments.

**Figure S6.** ADAM9 protein expression levels are higher in prostate cancer (PCa) tissues than normal prostate tissues. ADAM9 protein expression levels in PCa specimens (*n* = 160) and adjacent normal tissue samples or normal prostate tissue samples (*n* = 30) from a tissue microarray were measured by IHC staining.
