# Supplementary figures and images for: Stabilization of ADAM9 by N-α-acetyltransferase 10 protein contributes to promoting progression of androgen-independent prostate cancer
Source: Cell Death Dis. 2020 Jul 27;11(7):591. doi: 10.1038/s41419-020-02786-2 (PMC7385149; doi:10.1038/s41419-020-02786-2)

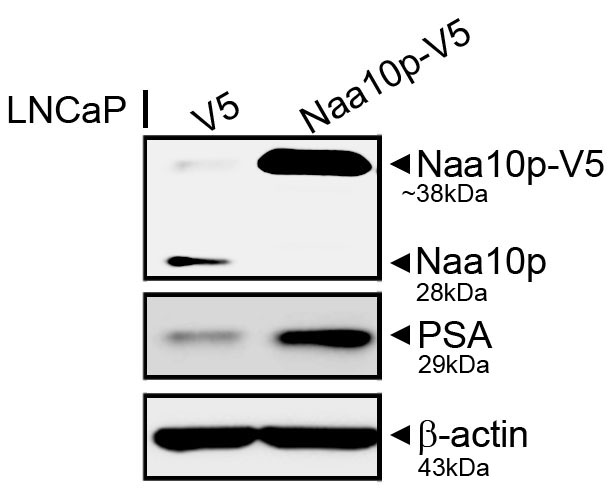

Supplement: Supplementary file 2 — Figure S1 [file 41419_2020_2786_MOESM2_ESM.tif]

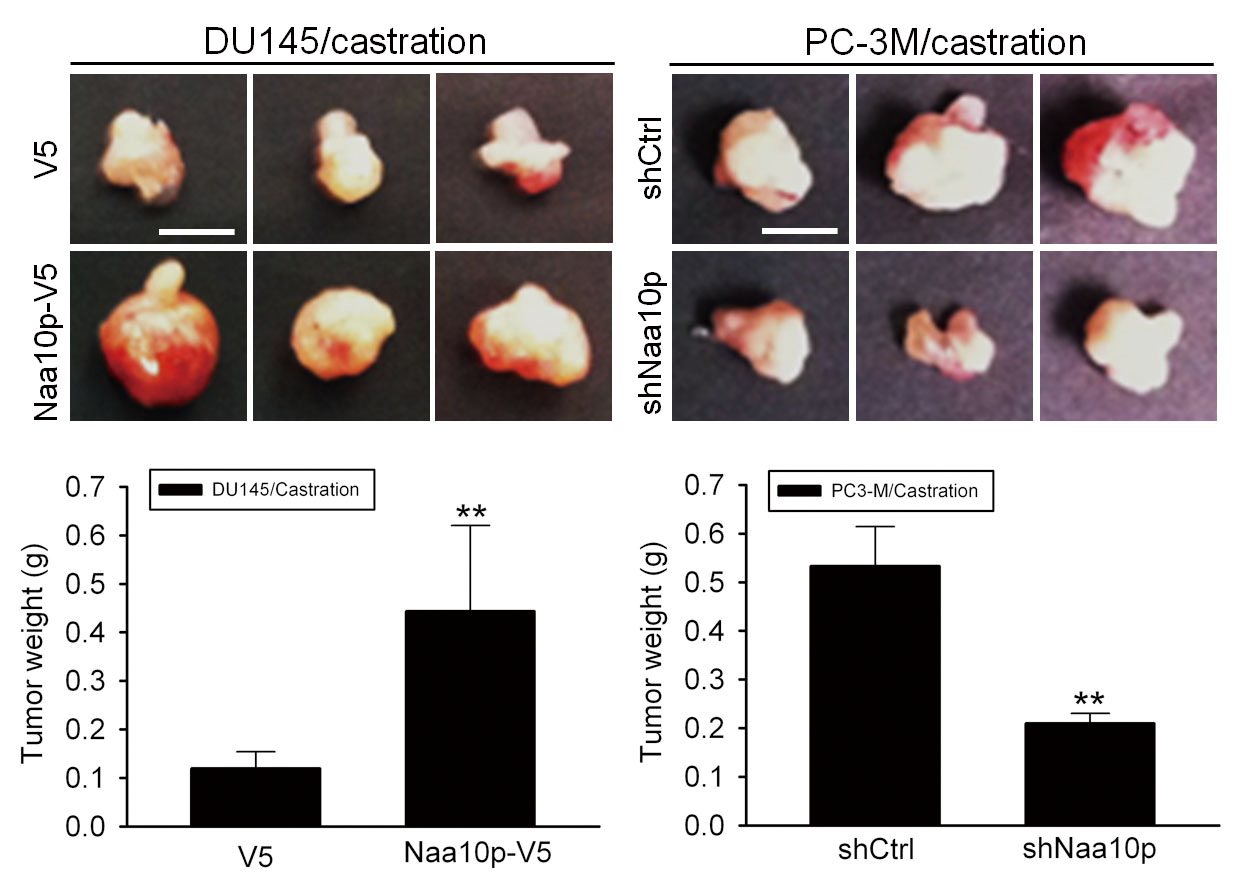

Supplement: Supplementary file 3 — Figure S2 [file 41419_2020_2786_MOESM3_ESM.tif]

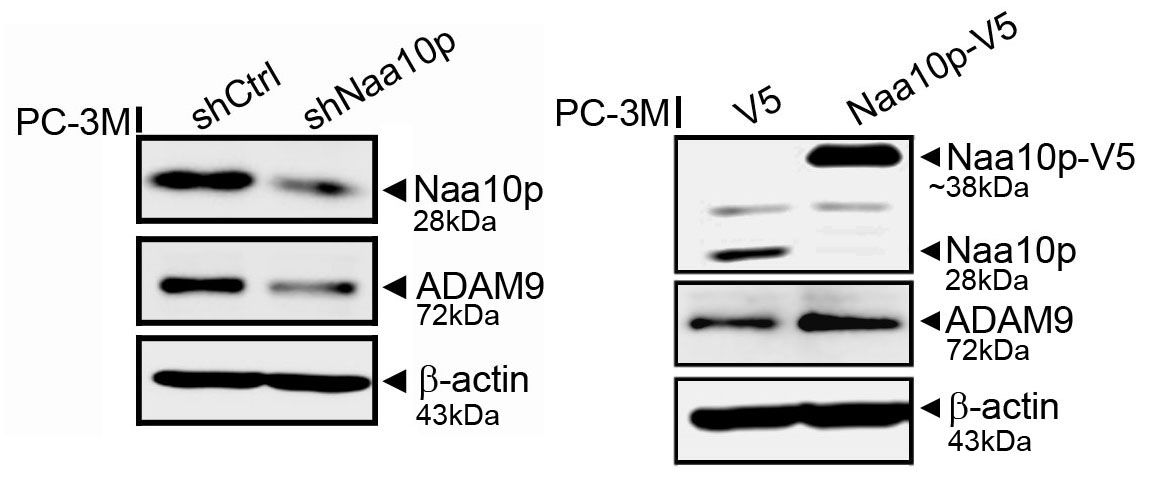

Supplement: Supplementary file 4 — Figure S3 [file 41419_2020_2786_MOESM4_ESM.tif]

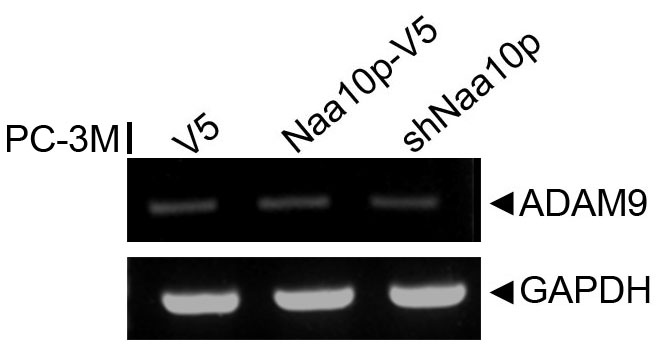

Supplement: Supplementary file 5 — Figure S4 [file 41419_2020_2786_MOESM5_ESM.tif]

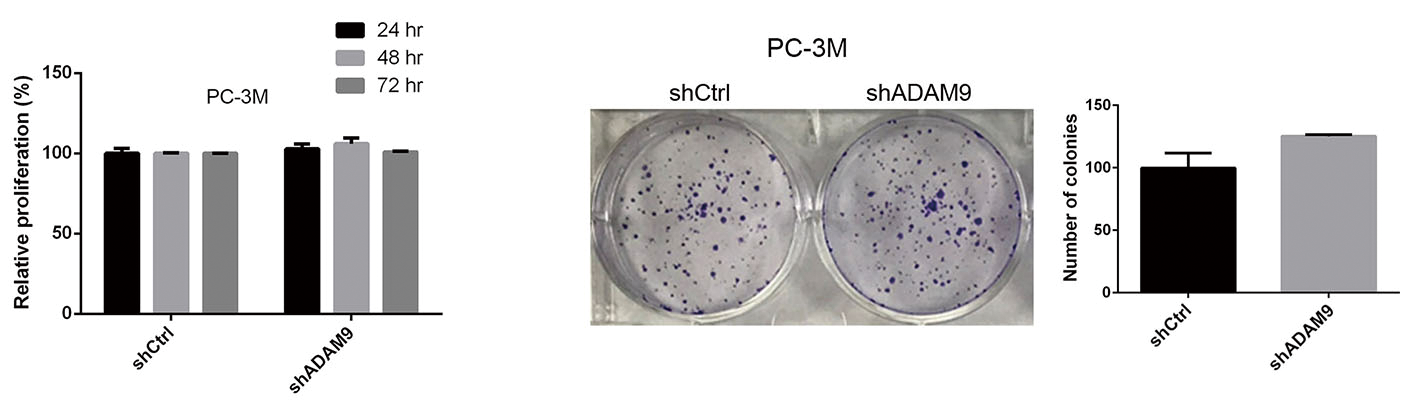

Supplement: Supplementary file 6 — Figure S5 [file 41419_2020_2786_MOESM6_ESM.tif]

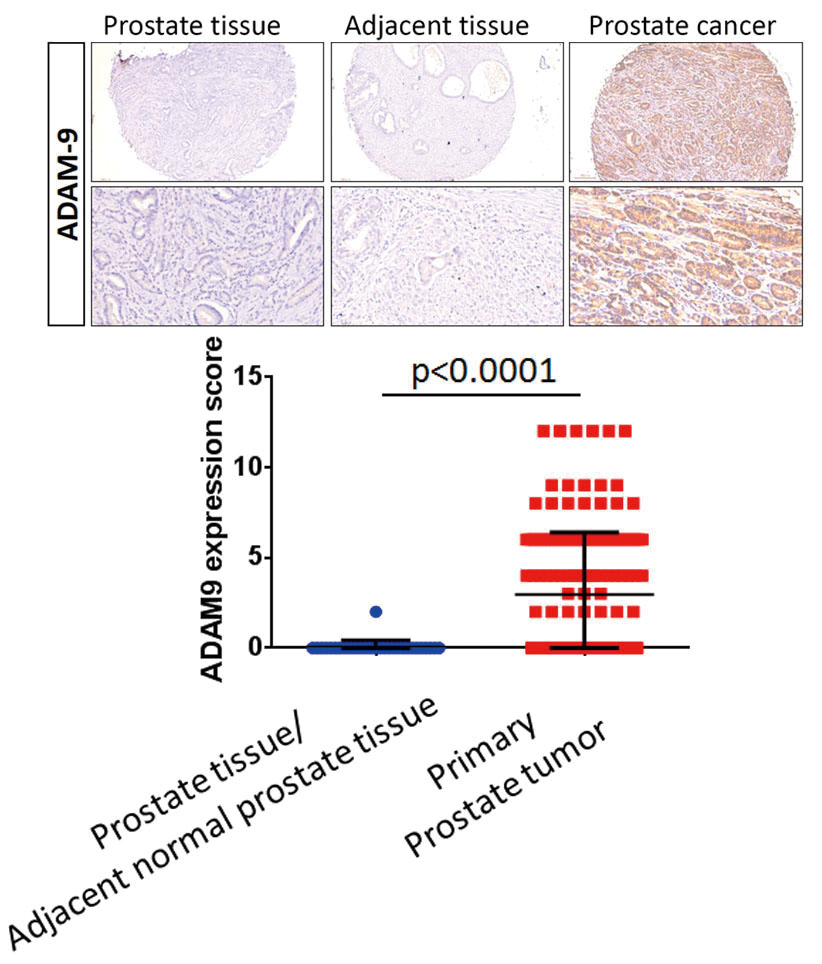

Supplement: Supplementary file 7 — Figure S6 [file 41419_2020_2786_MOESM7_ESM.tif]
